# Silymarin in Type 2 Diabetes Mellitus: A Systematic Review and Meta-Analysis of Randomized Controlled Trials

**DOI:** 10.1155/2016/5147468

**Published:** 2016-06-01

**Authors:** Luminita Voroneanu, Ionut Nistor, Raluca Dumea, Mugurel Apetrii, Adrian Covic

**Affiliations:** Nephrology Department, Faculty of Medicine, University of Medicine and Pharmacy “Gr. T. Popa”, 700503 Iasi, Romania

## Abstract

Type 2 diabetes mellitus (T2DM) is associated with increased risk of cardiovascular disease and nephropathy—now the leading cause of end-stage renal disease and dialysis in Europe and the United States. Inflammation and oxidative stress play a pivotal role in the development of diabetic complications. Silymarin, an herbal drug with antioxidant and anti-inflammatory properties, may improve glycemic control and prevent the progression of the complications. In a systematic review and meta-analysis including five randomized controlled trials and 270 patients, routine silymarin administration determines a significant reduction in fasting blood glucose levels (−26.86 mg/dL; 95% CI −35.42–18.30) and HbA1c levels (−1.07; 95% CI −1.73–0.40) and has no effect on lipid profile. Benefits for silymarin on proteinuria and CKD progressions are reported in only one small study and are uncertain. However, being aware of the low quality of the available evidence and elevated heterogeneity of these studies, no recommendation can be made and further studies are needed.

## 1. Introduction

Type 2 diabetes mellitus (T2DM) is one of the fastest growing health problems in the world, reaching epidemic proportion; globally it is estimated that 382 million people suffer from diabetes, that is, a prevalence of 8.3% [1]. T2DM is the fourth leading cause of death in developed countries, with a twofold excess mortality and twofold to fourfold increased risk of coronary heart disease and stroke [2]. About 20–30% of patients with diabetes develop evidence of nephropathy, now the leading cause of end-stage renal disease (ESRD) and dialysis in the US and in Europe [3]. Importantly, diabetes places large financial demands on the healthcare system: an estimated $245 billion in 2012 in the US, which is expected to rise with the increasing number of newly diagnosed individuals [4].

Managing diabetes is a considerable challenge to patients, providers, and healthcare systems all over the world. Better treatment options to reduce both the development and progression of diabetes complications are urgently required. In this context, it is important to search outside the field of conventional drugs and evaluate alternative medicine products for new treatments for diabetic nephropathy.

The extracts of milk thistle,* Silybum marianum*, have been considered as medical remedies since the time of ancient Greece and are now widely used as an alternative medication [5]. It is derived from* Silybum marianum* (milk thistle), an edible plant; it is native to the Mediterranean and grows all through Europe and North America and in India, China, South America, Africa, and Australia [5, 6]. The mechanisms of action of silymarin are not fully understood. Silymarin possesses antioxidant activity. It inhibits lipid peroxidation [7, 8], prevents glutathione depletion [9], and activates antioxidant enzymes that protect DNA from degradation [10]. These properties are determined largely by the presence of *β* ring catechol group (dihydroxylated *β*-ring) able to donate hydrogen electrons that stabilize radical species [11]; additionally, the presence of 2,3 unsaturation in conjugation with a 4-oxo-function in the Cring and the presence of functional groups capable of binding transition metal ions, such as iron, may also be responsible for the antioxidant nature of silymarin [12, 13]. In mice, silymarin administration determined a significant rise in pancreatic and plasma glutathione, prevented lipid peroxidation, and blunts the sustained increment in plasma glucose induced by alloxan [14]. Silymarin administration in streptozotocin treated rats increases the renal activity of several antioxidants enzymes, protecting the kidney from diabetic damage; it decreases podocyte superoxide generation in high glucose-induced models and in vivo in the kidney cortex [14]. Silymarin prevents the damage induced by oxidative agents in AKI [15]. It also prevented glomerular and tubular cell injury and apoptosis in cisplatin- and arsenic-treated rats reducing the ROS generation and apoptosis of tubular cells [16].

An anti-inflammatory effect of silymarin has been described in the liver tissue, in diabetes, or in experimental inflammatory bowel disease; there is evidence that silymarin regulates several inflammatory mediators such as tumoral necrosis factor-alpha (TNF-*α*), interleukin (IL-1*β*, IL-6, and IL-1) receptor antagonists, and nitric oxide. Moreover, silymarin downregulates prostaglandin and leukotriene synthesis, two powerful neutrophil chemoattractants, inhibits cyclooxygenase II, additionally reduces the cytotoxic activity and CD8 proliferation, and decreases neutrophil sequestration to the site of inflammation [17].

Oxidative stress and inflammation are considered as major alternative pathways contributing to the pathogenesis of diabetic nephropathy [18]. Silymarin administration in experimental diabetes induced in mice reduced levels of inflammatory cytokines (TNF-*α* and IL-1*β*) and oxidative stress mediators like myeloperoxidase activity, lipid peroxidation, carbonyl, and thiol content of pancreatic tissue in an almost dose-dependent manner [17]. In a small randomized controlled trial (RCT) including 60 patients with T2DM and diabetic nephropathy, silymarin reduced urinary and serum TNF-*α* level compared with placebo; additionally, a significant correlation was found between changes in urinary albumin-creatinine ratio (UACR) and urinary TNF-*α* level in silymarin-treated patients [19].

Additionally, silymarin possesses antifibrotic properties. It suppresses the expression of profibrogenic procollagen alpha 1 and tissue inhibitor of metalloproteinase-1 (TIMP-1), most likely via downregulation of transforming growth factor-beta 1 (TGF-*β*1) mRNA in rats with biliary fibrosis [20]. Moreover, it determines a significant reduction of TGF-*β* [21]. TGF-*β* plays a key role in the pathogenesis of diabetic nephropathy by mediating glomerulosclerosis and tubulointerstitial fibrosis [22]. It is already demonstrated that its urinary and serum levels are directly correlated with degree of proteinuria and progression of diabetic nephropathy [22]. Silymarin administration determined a reduction in urinary and serum levels of TGF-*β* in patients with T2DM [23, 24].

This systematic review focuses on the evidence related to silymarin use in diabetes, which is discussed in detail. Therefore, the aim of this meta-analysis was to establish more clearly the benefits of silymarin therapy in patients with diabetes.

## 2. Why It Is Important to Do the Review

Despite theoretical benefit and efficacy in culture cells of silymarin, a systematic review that included 14 studies found no clear benefits on mortality, improvement in liver histology, or improvements of biochemical markers of liver function in patients with chronic liver disease [25]. To the best of our knowledge there is no systematic review assessing the efficacy of silymarin in diabetes or in renal disease. A number of reviews of complementary and alternative medicine in diabetes were published. A systematic review of Chinese herbs used in T2DM has been published by the Cochrane Library [21], but it includes only one small study involving silymarin [26]. Although the meta-analysis by Suksomboon et al. [27] includes more trials and did not cover silymarin in its scope, the only outcome was glycemic control. This systematic review summarizes the available evidence from RCTs about the effects of silymarin in T2DM.

## 3. Search Methods for Review

Electronic databases, PubMed, MEDLINE, EMBASE, Cochrane Central Register of Controlled Trials (CENTRAL), AMED (Allied and Complementary Medicine Database), EBM Reviews, ACP Journal Club, and MD Consult, were searched using the terms: milk thistle,* Silybum marianum*,* Silybum*, silymarin, silibinin, silybin, silicristin, silidianin, spelling variants, and diabetes.

## 4. Types of Participants

### 4.1. Intervention

Adults (18 years or older) with T2DM were included. Intervention was considered to be included when silymarin based compounds were given. The control group includes placebo or standard care only (any active intervention used with the intention of lowering blood glucose levels, e.g., metformin, sulphonylureas, acarbose, and insulin). Silymarin plus other therapies such as other herbs (*Barberis*) was excluded. Trials were only included if the treatment was given for a minimum of one month. Cointerventions were allowed as long as both arms of the RCT received the same cointervention(s). Only randomized controlled trials were included.

## 5. Types of Outcome Measures

 Consider the following:

- Mortality (diabetes-related and all-cause).
- Diabetes complications (neuropathy, retinopathy, nephropathy, chronic kidney disease (CKD) progression, changes in eGFR, and changes in proteinuria).
- Glycemic control (glycated haemoglobin levels (HbA1c) and fasting blood glucose levels).
- Lipid control (changes in cholesterol and triglyceride).
- Adverse events.

## 6. Data Extraction and Management

Data extraction was carried out independently by two authors using standard data extraction forms. Where more than one publication of one study exists, reports were grouped together and the publication with the most complete data was used in the analyses. Where relevant outcomes are only published in earlier versions these data were used. Any discrepancy between published versions was highlighted. Risk of bias was assessed using standard domains (Higgins JPT, Green S (editors); Cochrane Handbook for Systematic Reviews of Interventions Version 5.1.0 [updated March 2011]; the Cochrane Collaboration, 2011, available from http://www.cochrane-handbook.org/). We summarized treatment effects using random-effects meta-analysis and expressed results as relative risks (RR) or rate ratios for binary outcomes (mortality, rate of fatal cardiovascular events, and rate of adverse events) or mean difference for continuous outcomes (fasting blood glucose levels, blood pressure, and changes in eGFR) together with 95% CIs. We assessed heterogeneity in treatment estimates using the Cochran *Q* test and *I* ^2^ statistic.

## 7. Results

### 7.1. Study Selection

**Figure 1:**
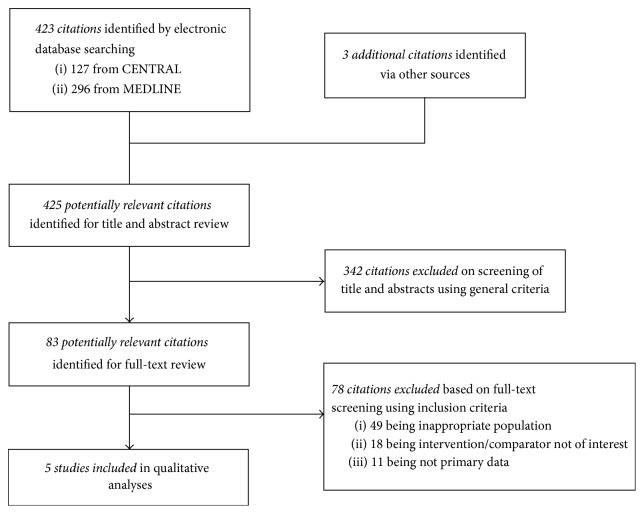
Selection and description of studies.

The electronic search identified 423 citations of which we excluded 342 studies based on title and abstract. After reading the full text of the remaining 83 citations we included in our final analysis 5 studies (RCTs) involving 270 patients (Figure 1).

### 7.2. Study Characteristics

Table 1 shows the key characteristics of the studies and patients included in our systematic review. Four studies evaluated only diabetic patients and one study included patients with diabetes and alcoholic cirrhosis. Follow-up ranged from 45 days to 6 months. Silymarin daily doses ranged between 200 and 600 mg. All studies evaluated short-term outcomes (glycemic control and lipid metabolism). Only one trial reported proteinuria, markers of inflammation and fibrosis while three trials reported malondialdehyde levels, a marker of oxidative stress. CKD progression (change in proteinuria or in creatinine levels or in eGFR were reported by only one study).

### 7.3. Risk of Bias of the Included Studies

#### 7.3.1. Selection Bias

**Figure 2:**
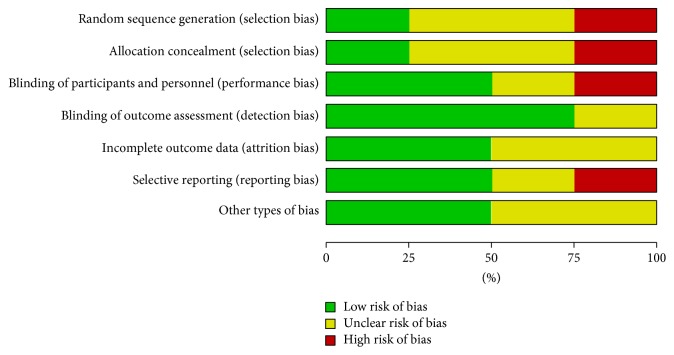
Risk of bias graph: review authors' judgments about each risk of bias item presented as percentages across all included studies.

**Figure 3:**
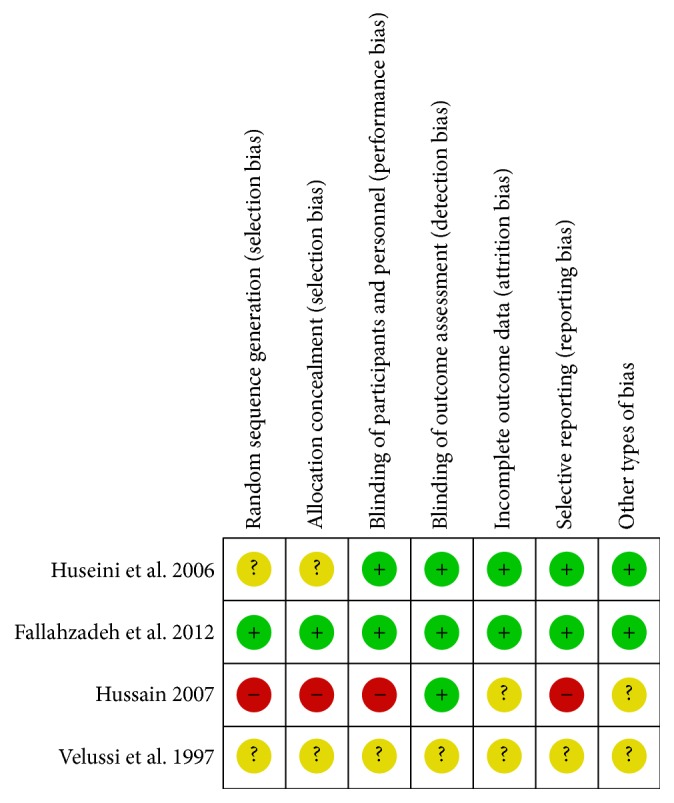
Risk of bias summary: review authors' judgments about each risk of bias item for each included study.

Two studies [19, 30] were at low risk of selection bias related to random sequence generation and allocation concealment as block randomization procedure (Random Allocation Software (RAS)) was used. Three studies [26, 28, 29] were unclear with respect to selection bias, as the methods used were not clearly described; see Figures 2 and 3.

#### 7.3.2. Detection and Performance Bias

The study by Velussi et al. 1997 [26] was an open control study with high risk of detection and performance bias, while the other included studies were using methods to blind the intervention. Three studies [19, 28, 30] were at low risk for performance bias since blinding was done, and one study [29] was judged at unclear risk for performance bias related to blinding, as the method used was not clearly described and did not report checking of blinding conditions.

Expected results were evaluated by laboratory blood and urine tests; in these circumstances we believe that the risk of bias is low in four studies [19, 28–30] in terms of detection bias. The study by Velussi et al. 1997 [26] is an open controlled study, and it was considered at a high risk for detection bias. No information about how and if the blinding was done was provided by the authors of the included studies.

### 7.4. Incomplete Outcome Data

Three studies had a low risk of attrition bias [28–30] as the primary and secondary outcomes were reported for the intention to treat population. By Velussi et al. 1997 [26] it was unclear how many patients were included in the final analysis.

### 7.5. Selective Reporting Bias

Selective reporting was at low risk in two studies [19, 28] as the main outcomes related to primary disease, stated in the protocol, were reported in the final manuscript. Three studies [26, 29, 30] were with a high risk of reporting bias, based on comparison of reported outcomes in the protocols and the main outcomes related to primary disease and side effects.

### 7.6. Other Potential Sources of Bias

Two studies [26, 29] were funded by pharmaceutical companies and therefore were judged to be at high risk of bias. For the other three studies we found no other source of bias.

### 7.7. Outcomes

#### 7.7.1. All-Cause Mortality

Only one study, with 60 patients, reported all-cause mortality [19]; there was only one death in the silymarin group; the cause of death was myocardial infarction probably due to underlying coronary artery disease and was not related to silymarin use.

#### 7.7.2. Diabetes Complications (Neuropathy, Retinopathy, and Nephropathy)

We found data only about diabetic nephropathy. One RCT, including 60 patients with T2DM with overt nephropathy, analyzed the efficacy and safety of adding silymarin to RAS inhibitors in reducing progression of diabetic nephropathy [19]. Mean values for changes in serum creatinine were not significantly different between the 2 groups (mean change in silymarin group: 0.021 (−0.027 to 0.07) and in the placebo group: 0.025 (−0.031 to 0.081); difference between groups: −0.004 (−0.076 to 0.069)). Similar results were reported also for eGFR: mean change in the silymarin group: −2.03 (−6.81 to 2.74) mL/min/1.73 m^2^ and in the placebo group: −1.81 (−5.75 to 2.14) mL/min/1.73 m^2^; difference between groups: −0.23 (−6.28 to 5.82) mL/min/1.73 m^2^.

Changes in proteinuria were also analyzed in this trial. Mean UACR levels decreased in both groups: −566 (−827 to −305) mg/g in the silymarin group versus −219 (−454 to 16) mg/g for placebo. However, this decrement was significantly higher in the silymarin group. Moreover, at the end of the treatment phase, UACR decreased more than 50% from baseline in 12 patients from the silymarin group compared with 6 patients from the placebo group (*P* = 0.09).

#### 7.7.3. Glycemic Control

**Figure 4:**
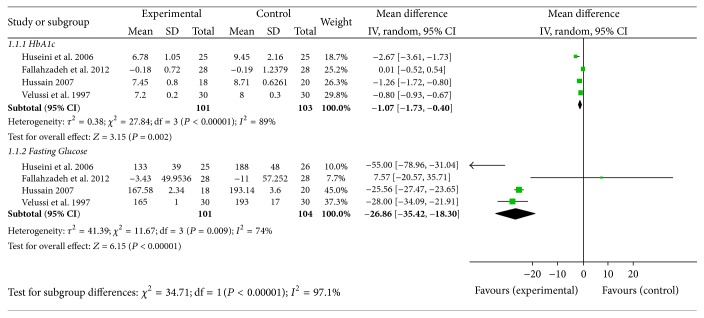
Glycemic control.

Silymarin administration was associated with a significant reduction in fasting blood glucose levels (mean difference [MD] (−26.86 mg/dL; 95% CI [−35.42, −18.30])) in four trials [19, 26, 28, 29]. Similarly, compared with placebo, silymarin administration reduced significantly HbA1c levels ([MD] 1.07; 95% CI [−1.73–0.40]); see Figure 4.

#### 7.7.4. Lipid Control

**Figure 5:**
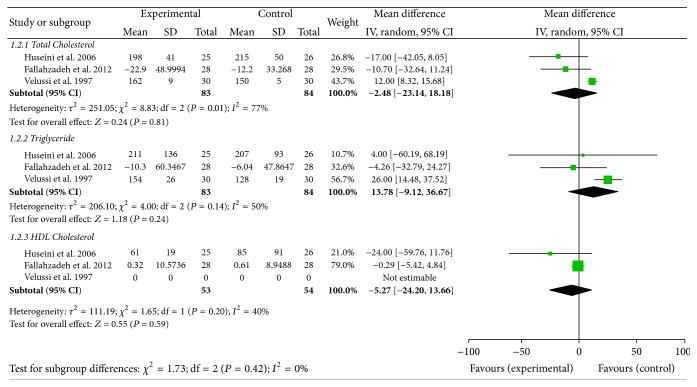
Lipid control.

Three studies reported data on this outcome [19, 26, 29]. No difference was found between the two arms: MD for cholesterol levels was −2.48 mg/dL; 95% CI −23.14–18.18; MD for HDL cholesterol was −5.27 mg/dL; 95% CI −24.20–13.66; MD for triglyceride was 13.87 mg/dL; 95% CI −9.12–36.67; see Figure 5.

#### 7.7.5. Adverse Events

Adverse events were reported only in two studies; except for the gastrointestinal disturbances and headache (data reported in one study), silymarin was found to be safe and without major side effects (see Table 2).

## 8. Discussion

In low- to very low-quality evidence from 5 RCTs trials done on 270 patients, routine silymarin administration in patients with T2DM might improve the glycemic control, has no effect on lipid profile, and has imprecise effects on CKD. Adverse effects were not reported systematically.

In the last ten years, silymarin was gradually recognized as hopeful complementary medication in diabetes. Silymarin treatment resulted in a statistically significant improvement in glycemic control in four studies compared with placebo. Heterogeneity was also observed in the study results. This may be due to differences in the dose of milk thistle used and in the treatment regimens. Besides, huge difference in baseline fasting blood glucose level may also play a part. However, although hyperglycemia was associated with an increased mortality and CV risk in epidemiological and pathophysiological studies in patients with T2DM [31], the association between the extent of glucose lowering and the reduction in CV risk is less well defined. Clinical trials evaluating the effect of intensive glycemic control on main outcomes in type 2 DM patients showed disappointing results. Results of the main Action to Control Cardiovascular Risk in Diabetes (ACCORD) trial indicate that a therapeutic strategy targeting HbA1c levels below 6.0% increased the rate of death from any cause (as compared with the standard-therapy group, the intensive-therapy group had a relative increase in mortality of 22% and an absolute increase of 1.0% during this follow-up period, with similar differences in death from CV causes) [32]. Moreover, results from a recently published secondary analysis of the ACCORD, including patients with mild-to-moderate CKD, were disappointing [33]. An intensive glycemic control was associated with a 41% increase in CV mortality and a 31% increase in all-cause mortality [33]. A recent systematic review with both meta-analysis and trial-sequential analysis of randomized clinical trials conducted by Hemmingsen et al. showed no meaningful reduction in major outcomes with intensive glycemic control in patients with type 2 DM [34]. In this meta-analysis (including 28,614 participants with type 2 DM from 20 RCTs), intensive glycemic control did not reduce all-cause mortality (RR 1.02; 95% CI 0.91–1.13). Additionally, it did not reduce the risk of CV mortality (RR 1.11; 95% CI 0.92–1.35). Intensive treatment reduced the risk for nonfatal MI (RR 0.85, 95% CI 0.76–0.95, and *P* = 0.004) in meta-analysis, but this was not confirmed in trial-sequential analysis. Furthermore, reduction in nephropathy was not significant (RR 0.83; 95% CI 0.64–1.06). Moreover, intensive control of blood glucose increases patients' relative risk of severe hypoglycemia by 30%. Patients with type 2 DM have a diversity of lipid abnormalities including high levels of chylomicron remnants, enlarged levels of LDL, and low levels of HDL [35]. Dyslipidaemia is a main risk factor for macrovascular complications in diabetes patients. Multiple clinical trials have showed favourable effects of lipid control on CVD outcomes in diabetic subjects with CHD and for primary CV prevention [36, 37]. A recent meta-analysis, including data from over 18,000 patients with diabetes from 14 randomized trials (mean follow-up 4.3 years), showed a 9% proportional reduction in all-cause mortality and 13% reduction in vascular mortality, for each mmol/L reduction in LDL cholesterol [37]. Treatment with silymarin did not successfully improve the lipid profile markers in our systematic review. Disparate data are provided by several experimental or human studies. Silymarin administration in rats with impaired lipid profile determines a significant reduction in LDL, VLDL, triglyceride, and cholesterol with elevation of HDL cholesterol [38]. Silymarin seems to decrease the intracellular cholesterol esterification (by diminishing acyl CoA enzyme activity). Silymarin inhibits HMG-CoA (3-hydroxy-3-methylglutaryl-coenzyme A) reductase enzyme and reduces the cholesterol synthesis [39]. Moreover, silymarin partially antagonizes the increase in liver content of triglycerides, decrease in VLDL synthesis, and the availability of free VLDL secretion in the intestine [40, 41]. Additionally, it reduces lipid accumulation by downregulating adipogenic factors, such as peroxisome proliferator-activated receptor *γ* (PPAR*γ*), CCAAT-enhancer binding protein *α* (C/EBP*α*), and fatty acid-binding protein 4 (FABP4) [42]. However, human studies showed different results. Several small human studies [40, 41] confirmed the benefit reported by experimental studies, while other small studies did not confirm a significant improvement in lipid metabolism after silymarin use.

Further studies are necessary before a firm conclusion can be made. No unquestionable data regarding the effects of silymarin on main outcome (mortality or progression of diabetic kidney disease) are available. Only one small study with a short duration of the treatment phase showed a reduction of proteinuria in patients with type 2 diabetes with overt nephropathy. Inhibition of inflammatory mediators and attenuation of oxidative stress may be the possible mechanisms behind this observed efficacy.

This systematic review has a number of potential limitations. First, the small number of studies with a small sample size and short-term follow-up limits the power of our meta-analyses. The different silymarin products (without specific details of formulations used) and different dosage regimens, treatment durations, and endpoints used also make drawing meaningful comparisons between studies difficult. Furthermore, it is not known how surrogate outcomes, such as glycemic control, can be translated into patient-relevant outcomes including progression to end-stage renal disease and mortality. This warrants further investigation.

## Figures and Tables

**Table 1:** Studies included in analysis.

Study	Type of study	Comparison	Study population	Follow-up	Inclusion criteria	Outcomes
Velussi et al. 1997 [26]	12-month open, controlled study	Silymarin plus standard therapy versus standard therapy alone	60 insulin-treated diabetics with alcoholic cirrhosis	4 mo	Age 45 to 70 years (i) NIDDM with alcoholic liver cirrhosis (ii) BMI < 29 kg/m^2^ (iii) Ascertained diabetes for a period of at least 5 years and being treated with insulin only (iv) Stable insulin therapy for a period of at least 2 years (v) Negative for markers of hepatitis A, hepatitis B, and hepatitis C and being not addicted to alcohol for a period of at least 2 years prior to the start of the study (vi) No bleeding from varices (vii) Liver biopsy (liver cirrhosis)	Fasting blood glucose Mean daily blood glucose levels Daily glycosuria levels HbA1c Malondialdehyde levels

Huseini et al. 2006 [28]	Randomized double-blind clinical trial	Silymarin plus conventional therapy versus placebo plus conventional therapy	51 patients with type 2 DM	4 mo	Type 2 diabetes according to ADA criteria (2003) (i) Age 40–65 years (ii) Having a fasting blood glucose level less than 250 mg/dL (iii) Duration of diabetes was more than 2 years, and their diabetes was not controlled exclusively by diet	Fasting blood glucose HbA1c Total cholesterol, LDL, and HDL and triglyceride GOT and GPT levels

Hussain 2007 [29]	Randomized, double-blind, placebo-controlled trial	silymarin + glibenclamide versus placebo + glibenclamide versus glibenclamide alone	59 patients with type 2 DM	4 mo	T2DM for at least 5 years (i) Already maintained on 10 mg/day glibenclamide and on diet control (ii) Fasting plasma glucose ≥ 10 mmol/L (iii) HbA1c ≥ 8% (iv) BMI ≥ 29 kg/m^2^	Fasting blood glucose HbA1c Body mass index (BMI)

Fallahzadeh et al. 2012 [19]	Randomized, double-blind, placebo-controlled, 2-arm parallel trial	Silymarin versus placebo	60 patients with type 2DM and macroalbuminuria	6 mo	Urinary albumin excretion >300 mg/24 h Treatment of hyperglycemia with (but not limited to) an oral hypoglycemic agent or insulin Treatment of hypercholesterolemia with a statin Presence of diabetic retinopathy	Absolute change in urinary albumin-creatinine ratio Urinary and serum levels of TNF-*α* Malondialdehyde and TGF*β*

Ebrahimpour Koujan et al. 2015 [30]	Randomized, triple-blinded, placebo-controlled clinical trial	Silymarin versus placebo	40 patients with type 2 DM	45 days	Type 2 diabetes patients (i) taking hypoglycaemic medications, (ii) having a body mass index (BMI) between 27 and 35 kg/m^2^, and (iii) following a stable habitual diet for the past three months	Antioxidant indices Superoxide dismutase (SOD) and glutathione peroxidase (GPX) activity and total antioxidant capacity C reactive protein

**Table 2:** Side effects.

Study	Silymarin group		*N*	Placebo		*N*
	Mean	SD		Mean	SD	
Velussi et al. 1997 [26]	2.16 ep/pac/an hypoglycemic event			2.2 ep/pac/an hypoglycemic event		
	No side effects					

Huseini et al. 2006 [28]	Not reported					

Hussain 2007 [29]	No side effects					

Fallahzadeh et al. 2012 [19]	Nausea and vomiting	3 (10%)	28	Nausea and vomiting	2 (6.7%)	28
	Headache	2 (6.7%)	28	Headache	0 (0%)	28
	Dyspepsia and bloating	1 (3.3%)	28	Dyspepsia and bloating	0 (0)	28

Ebrahimpour Koujan et al., 2015 [30]	No side effects					
